# The effect of heel inserts and foot orthoses in older people with plantar heel pain

**DOI:** 10.1186/1757-1146-4-S1-P6

**Published:** 2011-05-20

**Authors:** Daniel R Bonanno, Karl B Landorf, Hylton B Menz

**Affiliations:** 1 Department of Podiatry, Faculty of Health Sciences, La Trobe University, Bundoora, Victoria, 3086, Australia; 2 Musculoskeletal Research Centre, Faculty of Health Sciences, La Trobe University, Bundoora, Victoria, 3086, Australia

## Background

Plantar heel pain is one of the most common musculoskeletal conditions affecting the foot and it is commonly experienced by older adults. Contoured foot orthoses and some heel inserts have been found to be effective for plantar heel pain, however the mechanism by which they achieve their effects is largely unknown. The aim of this study was to investigate the effects of foot orthoses and heel inserts on plantar pressures in older adults with plantar heel pain.

## Methods

Thirty-six adults aged over 65 years with at least 4 weeks duration of plantar heel pain participated in the study. Using the in-shoe Pedar^®^ system, plantar pressure data was recorded while participants walked along an 8 metre walkway wearing a standardised shoe and 4 different shoe inserts. The shoe inserts consisted of a silicon heel cup, a soft foam heel pad, a heel lift and a prefabricated foot orthosis. Data were collected for the heel, midfoot and forefoot.

## Results

Statistically significant attenuation of heel peak plantar pressures was provided by 3 of the 4 shoe inserts. The greatest reduction was achieved by the prefabricated foot orthosis, which provided a fivefold reduction compared to the next most effective insert. The contoured nature of the prefabricated foot orthosis allowed for an increase in midfoot contact area, resulting in a greater redistribution of force. The prefabricated foot orthosis was also the only shoe insert that did not increase forefoot plantar pressures.

## Conclusions

These findings indicate that the prefabricated foot orthosis may be the most appropriate insert to use when a reduction in vertical loading of the heel is required.

